# Effects of high occupational physical activity, aging, and exercise on heart rate variability among male workers

**DOI:** 10.1186/s40557-015-0073-0

**Published:** 2015-09-25

**Authors:** Dongmug Kang, Youngki Kim, Jongeun Kim, Yongsik Hwang, Byungmann Cho, Taekjong Hong, Byungmok Sung, Yonghwan Lee

**Affiliations:** Department of Occupational and Environmental Medicine, Pusan National University Yangsan Hospital, Yangsan, Korea; Environmental Health Center of Asbestos, Pusan National University Yangsan Hospital, Yangsan, Korea; Medical Research Institute, Pusan National University Hospital, Busan, Korea; Department of Preventive & Occupational Medicine, Pusan National University School of Medicine, Yangsan, Korea; Department of Cardiology, Pusan National University Hospital, Busan, Korea; Department of Preventive Medicine, Kosin University Graduate School, Busan, Korea

**Keywords:** Cardiovascular disease, Exhaustion, Social support, Parasympathetic, Relative heart rate ratio, Vagal, Workload, Work limit

## Abstract

**Objectives:**

Effects of aging and leisure time physical activity (LPA) might influence the effect of occupational physical activity (OPA) on risk for cardiovascular disease (CVD). This study was conducted to determine whether OPA affects CVD after controlling the effects of LPA and other risk factors for CVD such as job stress.

**Methods:**

Participants were 131 male Korean manual workers. Tests for heart rate variability (HRV) were conducted for five minutes in the morning at work. We defined OPA as the combined concept of relative heart rate ratio (RHR), evaluated using a heart rate monitor.

**Results:**

Whereas high OPA was not related to any HRV items in the younger age group, high OPA was associated with an increased number of low-value cases among all HRV items in older workers. Exercise had beneficial effects only in the younger group. After controlling for exercise and other risk factors, the odds ratios of the root-mean square of the difference of successive normal R-R intervals (rMSSD) and high frequency band power (HF) among the older age and high OPA group compared with the younger age and low OPA group were 64.0 and 18.5, respectively. Social support and shift work were independent risk factors in HRV.

**Conclusions:**

OPA in aging workers increases CVD risks. This study provides support for the need for protection of aging workers from physical work overload, and indicates the need for further study of optimal limits of OPA.

## Introduction

The global burden of cardiovascular disease (CVD) is extremely high, with CVD being involved in approximately one-third of all deaths worldwide [1]. One way to reduce the risk of CVD is through physical activity: both leisure time physical activity (LPA) and occupational physical activity (OPA) are thought to reduce cardiovascular risk by decreasing incidence of coronary heart disease and stroke [2, 3]. Although there is support for the conclusion that OPA is beneficial for CVD, the evidence is limited to mild to moderate OPA [2, 4, 5]. With respect to high OPA, previous studies have shown conflicting results ranging from protective [2] to no effects on CVD [4], and even some adverse consequences on cardiovascular health [5].

The burden of OPA on an individual worker varies according to their physical work capacity (PWC) [6]. PWC is largely dependent on age, and PWC has been shown to decrease rapidly after the age of 45 [7]. While the evidence suggests that the effects of OPA on health would differ for groups under and over age 45 years, there are currently no studies that directly compare the different effects of OPA on these two age groups. Given that LPA has also been shown to affect an individual’s cardiovascular health, it is conceivable that LPA and OPA might both have varying effects on health according to age. While a previous study reported on the paradoxical effects of OPA and LPA on health, no prior investigations have considered the importance of aging on OPA and LPA related health changes [8].

Heart rate variability (HRV) is valuable non-invasive tool that can be used to check the balance of the autonomic nervous system in the heart [9, 10]. As HRV is inversely related to hypercholesterolemia [11], hypertension [12], coronary atherosclerosis [13], and stroke [14], HRV can be regarded as a preclinical marker for CVDs. Although several studies have reported the effects of job stress on HRV [15, 16], few have addressed the relationship between HRV and high OPA. Additionally, while other occupational risk factors for HRV have been investigated including the presence of lead [17], manganese [18], and work shift, as well as general risk factors such as smoking [19] no studies focusing on OPA have adjusted for these risk factors.

The purposes of the current study was to evaluate the effects of high OPA on HRV according to age, investigate the different effects of OPA on HRV in relation to LPA, and evaluate the effects of high OPA on HRV after controlling for LPA and other risk factors for HRV.

## Materials and methods

### Study population

Five companies participated in this research, which was conducted from June 2003 to May 2004. One occupational physician and one industrial hygienist used a survey to select study participants from a group of manual workers, who were recruited based on similar OPA levels. The companies included foundry work(total manual workers: *n* = 169, study participants: *n* = 21), ship-building (total manual workers: *n* = 987, study participants: *n* = 46), car engine assembly (total manual workers: *n* = 489, study participants: *n* = 84), ship engine manufacture (total manual workers: *n* = 321, study participants: *n* = 30), and railway vehicle manufacture (total manual workers: *n* = 474, study participants: *n* = 43). The total number of participants in the study was 224; all participants were male. We exclude77 participants who did not undergo the heart rate monitor (HRM) evaluation that was used to assess OPA. Because the study was focused on occupational physical factors, we excluded participants with known risk for HRV, such as hypertension or diabetes mellitus patients (*n* = 7), and contact with metal fumes or organic solvents exposure cases (*n* = 9) to control for manganese and lead exposure. Before the study began, written informed consents were obtained from all participants. Ethical concerns for the current study were reviewed by the medical research institute of Pusan National University Hospital.

### Occupational physical activity

OPA was checked using HRM (Polar Electro Co, Finland, S810^TM^). HRMs were attached to the participants’ chest during work. A work diary was also implemented, which was checked by researchers to determine daily work time and rest time. Relative heart ratio (RHR) was calculated using the following equation:

$$ \mathrm{R}\mathrm{H}\mathrm{R}\ \left(\%\right) = \left(\mathrm{H}\mathrm{R}\ \mathrm{during}\ \mathrm{work}\ \hbox{--}\ \mathrm{H}\mathrm{R}\ \mathrm{during}\ \mathrm{rest}\right)\ \mathrm{x}\ 100/\left(\mathrm{HR} \max \hbox{--}\ \mathrm{H}\mathrm{R}\ \mathrm{during}\ \mathrm{rest}\right) $$

Maximum heart rate (HRmax) was obtained using a cycle ergometer (Combi Co., Korea, Aerobike 75XL II®). We defined OPA as a combination of RHR and number of working hours per day, as suggested by Wu and Wang [20]. OPA was divided into high and low as follows: high OPA was defined as RHR ≥ 16.0 (%) with work time ≥ 12 h, or 16.0 (%) ≤ RHR < 20 (%) with 10 h ≤ work time < 12 h, or 20.0 (%) ≤ RHR < 24.5 (%) with 8 h ≤ work time < 10 h, or RHR ≥ 24.5 (%) with work time > 8 h. All other combinations of RHR and work time were defined as low OPA.

### Heart rate variability

All participants were required to refrain from drinking alcohol one day before the HRV test. Tobacco smoking and coffee drinking were also prohibited 30 min before HRV testing. Tests were conducted in a noise-free environment from 9 ~ 10 A.M. after a 30-min rest. HRV was tested for five minutes using the SA-2000E (Medicore, Korea, SA-2000E) according to the guidelines of the task force of the European Society of Cardiology and the North American Society of Pacing and Electrophysiology (20). Data were divided into time domain and frequency domain. Time domain was composed of the standard deviation of the normal to normal (NN) interval (SDNN), the square root of the mean squared differences of successive NN intervals (rMSSD), and the proportion of successive NN intervals greater than 50 ms (pNN50%). The frequency domain was composed of very low-frequency spectral power (VLF), total spectral power (TP), low-frequency power (LF), high-frequency power (HF), and LF/HF ratio in both ms^2^ and normalized units. Because the Task Force recommends not using pNN50, TP, and VLF from short-term measures, we used SDNN, rMSSD, LF, HF, and LF/HF ratio for the final analyses [21]. Because low HRV is associated with poor health, we divided HRV results into low and normal groups using proposed reference values for short-term HRV [22].

### Questionnaire

A structured questionnaire was administered before the HRM and HRV tests. Demographics, health behavior including frequency of exercise during weekdays representing LPA, and work-related variables were assessed. The Korean version of the Job Content Questionnaire (JCQ) was used to check job stress. Validity and reliability of the Korean version of the JCQ have been evaluated elsewhere [23]. The Chronbach’s alpha values for job demand, decision latitude, and social support were 0.66, 0.59, and 0.86 in the current study, respectively. Each items of the JCQ were dichotomized by the median score.

### Statistical analysis

Statistical analyses were conducted using SAS (version 9.3), with a significance level 0.05. Missing data for each variable were not considered in the analyses. Dependent measures were the dichotomous variables (low and normal) for each HRV items. Cochran Armitage trend tests were conducted to assess the relationship between categorical variables. Statistical analyses with a null value in the column position or raw total could not be conducted (identified as “not applicable”). After evaluation of the relationship between age, OPA, and exercise (LPA), multiple logistic regressions were used to adjust for exercise and other risk factors. Statistically significant variables related to HRV in univariate analyses were then used in multiple logistic regressions. To identify the combined effect of aging and OPA, four groups were used in the multiple logistic regressions: younger age and low OPA, younger age and high OPA, older age and low OPA, and older age and high OPA. Due to small numbers in some of the categories, data reduction by merging groups of variables was conducted for logistic regression.

## Results

### Relationship between demographic, health, and work-related variables with HRV

The results of trend tests between variables and time domain of HRV items are listed below. Older age group and lower social support were associated with increased cases of low SDNN. For frequency domain, older age and heavier alcohol use had more instances of low LF. Older age, higher body mass index (BMI), heavier alcohol drinking, and shift workers, and lower social support groups had more cases of low LF:HF ratio. However, lower social support was associated with fewer instances of low LF:HF ratio (Table 1).

**Table 1:** Results of trend tests between heart rate variability items and variables

		SDNN				rMSSD				LF				HF				LF:HF			
		low		normal		low		normal		low		normal		low		normal		low		normal	
		n	%	n	%	n	%	n	%	n	%	n	%	n	%	n	%	n	%	n	%
Age (year)	< 45	24	21.2	89	78.8	7	6.19	106	93.8	29	25.7	84	74.3	12	10.6	101	89.4	35	31	78	69.0
	≥45	8	44.4	10	55.6	3	16.7	15	83.3	9	50	9	50	4	22.2	14	77.8	10	55.6	8	44.4
	*p*-value	0.0167				0.0601				0.0173				0.0813				0.0207			
OPA	Low	20	21.7	72	78.3	5	5.4	87	94.6	23	25.0	69	75.0	9	9.8	83	90.2	32	34.8	60	65.2
	High	12	30.8	27	69.2	5	12.8	34	87.2	15	38.5	24	61.5	7	18.0	32	82.1	13	33.3	26	66.7
	*p*-value	0.1357				0.0727				0.0603				0.0959				0.4366			
Exercise (times/ week)	≥3	9	24.3	28	75.7	2	5.4	35	94.6	12	32.4	25	67.6	4	10.8	33	89.2	16	43.2	21	56.8
	1–2	14	23.3	46	76.7	4	6.7	56	93.3	16	26.7	44	73.3	6	10.0	54	90.0	21	35.0	39	65.0
	0	6	27.3	16	72.7	4	18.2	18	81.8	8	36.4	14	63.6	4	18.2	18	81.8	6	27.3	16	72.7
	*p*-value	0.4199				0.0600				0.4384				0.2343				0.1036			
BMI (kg/m^2^)	< 20	1	14.3	6	85.7	0	0.0	7	100.0	2	28.6	5	71.4	0	0.0	7	100.0	2	28.6	5	71.4
	20–25	25	24.5	77	75.5	8	7.8	94	92.2	31	30.4	71	69.6	14	13.7	88	86.3	31	30.4	71	69.6
	> 25	6	27.3	16	72.7	2	9.1	20	90.9	5	22.7	17	77.3	2	9.1	20	90.9	12	54.6	10	45.5
	*p*-value	0.2758				0.2688				0.2843				0.4609				0.0253			
Smoking	Present	13	28.3	33	71.7	5	10.9	41	89.1	14	30.4	32	69.6	7	15.2	39	84.8	18	39.1	28	60.9
	Previous	4	25.0	12	75.0	2	12.5	14	87.5	6	37.5	10	62.5	3	18.8	13	81.3	6	37.5	10	62.5
	No	12	21.1	45	79.0	3	5.3	54	94.7	16	28.1	41	71.9	4	7.0	53	93.0	19	33.3	38	66.7
	*p*-value	0.1980				0.1483				0.3874				0.0935				0.2699			
Alcohol drinking (times/week)	≥2	19	21.6	69	78.4	7	8.0	81	92.1	21	23.9	67	76.1	10	11.4	78	88.6	26	29.6	62	70.5
	< 2	13	30.2	30	69.8	3	7.0	40	93.0	17	39.5	26	60.5	6	14.0	37	86.1	19	44.2	24	55.8
	*p*-value	0.1399				0.4216				0.0317				0.3354				0.0488			
Shiftwork	Yes	14	23.7	45	76.3	4	6.8	55	93.2	19	32.2	40	67.8	6	10.2	53	89.8	30	50.9	29	49.2
	No	15	25.0	45	75.0	6	10.0	54	90.0	17	28.3	43	71.7	8	13.3	52	86.7	13	21.7	47	78.3
	*p*-value	0.4359				0.2633				0.3229				0.2961				0.0005			
Job demand	Low	16	25.4	47	74.6	4	6.4	59	93.7	19	30.2	44	69.8	7	11.1	56	88.9	25	39.7	38	60.3
	High	12	23.5	39	76.5	5	9.8	46	90.2	15	29.4	36	70.6	6	11.8	45	88.2	16	31.4	35	68.6
	*p*-value	0.4089				0.2482				0.4655				0.4565				0.1790			
Decision latitude	Low	15	22.7	51	77.3	6	9.1	60	90.9	18	27.3	48	72.7	7	10.6	59	89.4	25	37.9	41	62.1
	High	13	26.5	36	73.5	3	6.1	46	93.9	17	34.7	32	65.3	6	12.2	43	87.8	15	30.6	34	69.4
	*p*-value	0.3192				0.2789				0.1962				0.3919				0.2092			
Social support	Low	21	30.4	48	69.6	6	8.7	63	91.3	23	33.3	46	66.7	8	11.6	61	88.4	20	29.0	49	71.0
	High	7	14.9	40	85.1	4	8.5	43	91.5	12	25.5	35	74.5	6	12.8	41	87.2	21	44.7	26	55.3
	*p*-value	0.0274				0.4861				0.1844				0.4246				0.0413			

*SDNN* the standard deviation of normal-to-normal intervals, *rMSSD* the root-mean square of the difference of successive normal R-R intervals, *LF* low frequency band power, *HF* high frequency band power, *LF:HF* ratio of low-frequency power to high-frequency power, *OPA* occupational physical activity, *BMI* body mass index

### Effects of OPA on HRV according to age and exercise respectively

To evaluate whether OPA effects on HRV differs between younger and older workers, we conducted trend tests between OPA and HRV items by each age group. In younger participants, no HRV items were associated with OPA. In older individuals, higher OPA groups had significantly more cases of low SDNN, rMSSD, HF, and HF/LF ratio. Trend tests between OPA and HRV by exercise showed inconsistent results: in the high exercise group, high OPA was significantly linked to greater instances of low rMSSD significantly. However, in the no exercise group, individuals with high OPA had significantly more cases of low LF, HF, and LF:HF ratio significantly (Table 2).

**Table 2:** Results of trend tests between heart rate variability items and occupational physical activity according to age and exercise frequency

			SDNN				rMSSD				LF				HF				LF:HF			
			low		normal		low		normal		low		normal		low		normal		low		normal	
			n	%	n	%	n	%	n	%	n	%	n	%	n	%	n	%	n	%	n	%
Age (years)		OPA																				
	< 45	Low	16	20.3	63	79.8	5	6.3	74	93.7	18	22.8	61	77.2	8	10.1	71	89.9	23	29.1	56	70.9
		High	8	23.5	26	76.5	2	5.9	32	94.1	11	32.4	23	67.7	4	11.8	30	88.2	12	35.3	22	64.7
		*p*-value	0.3481				0.464				0.1428				0.3977				0.2573			
	≥45	Low	4	30.8	9	69.2	0	0.0	13	100.0	5	38.5	8	61.5	1	7.7	12	92.3	9	69.2	4	30.8
		High	4	80.0	1	20.0	3	60.0	2	40.0	4	80.0	1	20.0	3	60.0	2	40.0	1	20.0	4	80.0
		*p*-value	0.0299				0.0011				0.0572				0.0084				0.0299			
Exercise (times/ week)																						
	≥ 3	Low	6	24.0	19	76.0	0	0.0	25	100.0	8	32.0	17	68.0	2	8.0	23	92.0	10	40.0	15	60.0
		High	3	25.0	9	75.0	2	16.7	10	83.3	4	33.3	8	66.7	2	16.7	10	83.3	6	50.0	6	50.0
		*p*-value	0.4735				0.0179				0.4677				0.2134				0.2827			
	1–2	Low	9	20.9	34	79.1	3	7.0	40	93.0	11	25.6	32	74.4	5	11.6	38	88.4	15	34.9	28	65.1
		High	5	29.4	12	70.6	1	5.9	16	94.1	5	29.4	12	70.6	1	5.9	16	94.1	6	35.3	11	64.7
		*p*-value	0.2420				0.4391				0.3812				0.2519				0.4880			
	0	Low	4	25.0	12	75.0	2	12.5	14	87.5	4	25.0	12	75.0	1	6.3	15	93.8	6	37.5	10	62.5
		High	2	33.3	4	66.7	2	33.3	4	66.7	4	66.7	2	33.3	3	50.0	3	50.0	0	0.0	6	100.0
		*p*-value	0.3479				0.1296				0.0352				0.0089				0.0393			

*SDNN* the standard deviation of normal-to-normal intervals, *rMSSD* the root-mean square of the difference of successive normal R-R intervals, *LF* low frequency band power, *HF* high frequency band power, *LF:HF* ratio of low-frequency power to high-frequency power, *OPA* occupational physical activity

### Effects of exercise on HRV according to age

Trend tests between exercise and HRV by age group revealed that among younger individuals, those who got less exercise had more cases of low rMSSD and HF. However, there was no association between exercise and HRV items in older participants (Table 3).

**Table 3:** Results of trend tests between heart rate variability items and exercise frequency per week according to age category

		SDNN				rMSSD				LF				HF				LF:HF			
Age (year)	Exercise (times / week)	low		normal		low		normal		low		normal		low		normal		low		normal	
		n	%	n	%	n	%	n	%	n	%	n	%	n	%	n	%	n	%	n	%
< 45	≥3	5	18.5	22	81.5	0	0.0	27	100.0	7	25.9	20	74.1	1	3.7	26	96.3	11	40.7	16	59.3
	1–2	10	18.9	43	81.1	3	5.7	50	94.3	12	22.6	41	77.4	5	9.4	48	90.6	17	32.1	36	67.9
	0	6	28.6	15	71.4	4	19.1	17	81.0	8	38.1	13	61.9	4	19.1	17	81.0	5	23.8	16	76.2
	*p*-value	0.2112				0.0059				0.197				0.0406				0.1061			
≥45	≥3	4	40.0	6	60.0	2	20.0	8	80.0	5	50.0	5	50.0	3	30.0	7	70.0	5	50.0	5	50.0
	1–2	4	57.1	3	42.9	1	14.3	6	85.7	4	57.1	3	42.9	1	14.3	6	85.7	4	57.1	3	42.9
	0	0	0.0	1	100.0	0	0.0	1	100.0	0	0.0	1	100.0	0	0.0	1	100.0	1	100.0	0	0.0
	*p*-value	0.5000				0.2994				0.3474				0.1727				0.2150			

*SDNN* the standard deviation of normal-to-normal intervals, *rMSSD* the root-mean square of the difference of successive normal R-R intervals, *LF* low frequency band power, *HF* high frequency band power, *LF:HF* ratio of low-frequency power to high-frequency power

### Effects of OPA on HRV according to age and exercise combined

To evaluate whether OPA effects on HRV differed according to age and exercise, trend tests between OPA and HRV items by each age and exercise category were conducted. In younger individuals, those with no exercise and high OPA had more cases of low LF and HF. In older individuals, exercise frequency did not seem to influence the effects of OPA on HRV. In the old age and frequent exercise group (≥3 times per week), high OPA was associated with more instances of low SDNN, rMSSD, and HF. Due to small sample size, only HF/LF ratio reached significance in the old age and moderate exercise group (1-2 times per week) with high OPA. In the old age and no exercise group, statistical analyses could not be conducted due to null values in the raw total column. Notably, there were no workers in the old age and no exercise group who had high OPA (Table 4).

**Table 4:** Results of trend tests between heart rate variability items and occupational physical activity according to combined category of age and exercise

			SDNN				rMSSD				LF				HF				LF:HF			
Age (year)	Exercise (frequency / week)	OPA	low		normal		low		normal		low		normal		low		normal		low		normal	
			n	%	n	%	n	%	n	%	n	%	n	%	n	%	n	%	n	%	n	%
< 45	≥3	Low	4	23.5	13	76.5	0	0.0	17	100.0	5	29.4	12	70.6	1	5.9	16	94.1	6	35.3	11	64.7
		High	1	10.0	9	90.0	0	0.0	10	100.0	2	20.0	8	80.0	0	0.0	10	100.0	5	50.0	5	50.0
		*p*-value	0.1911				na				0.2950				0.2172				0.2263			
	1–2	Low	7	18.0	32	82.1	3	7.7	36	92.3	9	23.1	30	76.9	5	12.8	34	87.2	11	28.2	28	71.8
		High	3	21.4	11	78.6	0	0.0	14	100.0	3	21.4	11	78.6	0	0.0	14	100.0	6	42.9	8	57.1
		*p*-value	0.3876				0.1427				0.4497				0.0796				0.1568			
	0	Low	4	26.7	11	73.3	2	13.3	13	86.7	4	26.7	11	73.3	1	6.7	14	93.3	5	33.3	10	66.7
		High	2	33.3	4	66.7	2	33.3	4	66.7	4	66.7	2	33.3	3	50.0	3	50.0	0	0.0	6	100.0
		*p*-value	0.3800				0.1458				0.0441				0.0112				0.0526			
≥45	≥3	Low	2	25.0	6	75.0	0	0.0	8	100.0	3	37.5	5	62.5	1	12.5	7	87.5	4	50.0	4	50.0
		High	2	100.0	0	0.0	2	100.0	0	0.0	2	100.0	0	0.0	2	100.0	0	0.0	1	50.0	1	50.0
		*p*-value	0.0264				0.0008				0.0569				0.0079				0.5000			
	1–2	Low	2	50.0	2	50.0	0	0.0	4	100.0	2	50.0	2	50.0	0	0.0	4	100.0	4	100.0	0	0.0
		High	2	66.7	1	33.3	1	33.3	2	66.7	2	66.7	1	33.3	1	33.3	2	66.7	0	0.0	3	100.0
		*p*-value	0.3296				0.1062				0.3296				0.1062				0.0041			
	0	Low	0	0.0	1	100.0	0	0.0	1	100.0	0	0.0	1	100.0	0	0.0	1	100.0	1	100.0	0	0.0
		High	0	0.0	0	0.0	0	0.0	0	0.0	0	0.0	0	0.0	0	0.0	0	0.0	0	0.0	0	0.0
		*p*-value	na				na				na				na				na			

*SDNN* the standard deviation of normal-to-normal intervals, *rMSSD* the root-mean square of the difference of successive normal R-R intervals, *LF* low frequency band power, *HF* high frequency band power; *LF:HF* ratio of low-frequency power to high-frequency power, *OPA* occupational physical activity; na: not applicable

### Combined effects of OPA and age on HRV adjusting exercise and other risk factors

After controlling for exercise and other risk factors in the univariate analysis, younger workers with low OPA were used as a reference group with which to compare the other three groups. Odds ratio (OR) of rMSSD and HF of the older age and high OPA group compared with the younger age and low OPA group were 64.0 and 18.5, respectively. Exercise had a protective effect on rMSSD with an OR of 0.2. Social support had protective effect on SDNN with OR of 0.3. BMI and shiftwork were independent risk factors for the HF/LF ratio. In a multiple logistic analysis, social support was not significantly related to the LF:HF ratio (Table 5).

**Table 5:** Multiple logistic regressions between heart rate variability items (low *vs.* normal) and age and occupational physical activity adjusting exercise and other covariates

		SDNN			rMSSD			LF			HF			LF:HF		
Covariates		OR	95% CI		OR	95% CI		OR	95% CI		OR	95% CI		OR	95% CI	
			lower	higher		lower	higher		lower	higher		lower	higher		lower	higher
Age and OPA																
	Age < 45 and low OPA	1.0			1.0			1.0			1.0			1.0		
	Age < 45 and high OPA	0.6	0.2	1.7	0.6	0.1	3.3	0.7	0.3	1.7	0.6	0.1	2.3	1.1	0.4	2.9
	Age ≥ 45and lower OPA	1.9	0.4	9.5	<0.1	<0.1	>99.9	1.9	0.4	8.5	1.2	0.1	12.5	3.1	0.6	16.6
	Age ≥ 45and high OPA	10.0	0.7	136.0	64.0	2.9	>99.9	4.2	0.3	51.4	18.5	1.3	259.8	<0.1	<0.1	>99.9
Exercise (frequency / week)																
	< 3	1.0			1.0			1.0			1.0			1.0		
	≥3	0.7	0.4	1.4	0.2	0.0	0.8	0.7	0.4	1.4	0.5	0.2	1.3	0.9	0.5	1.8
Body mass index (kg/m^2^)																
	Normal (20 - 25)													1.0		
	Abnormal													3.1	1.1	8.6
Alcohol drinking (frequency / week)																
	<2							1.0						1.0		
	≥2							1.7	0.7	4.2				1.4	0.5	3.6
Shift work																
	No													1.0		
	Yes													3.4	1.2	9.2
Social support																
	Low	1.0												1.0		
	High	0.3	0.1	0.9										1.9	0.8	4.5

*SDNN* the standard deviation of normal-to-normal intervals, *rMSSD* the root-mean square of the difference of successive normal R-R intervals, *LF* low frequency band power, *HF* high frequency band power, *LF:HF* ratio of low-frequency power to high-frequency power, *CI* confidence interval, *OPA* occupational physical activity

## Discussions

In the current study, the effects of OPA on HRV significantly differed according to age. Whereas high OPA was not related with any HRV items in the younger age group, high OPA increased the occurrence of low cases in all HRV items except LF (borderline significance) in older participants. After adjusting for exercise and other risk factors by multiple logistic regression analysis, high OPA in older individuals compared with low OPA in younger individuals was related to an increase in the number of cases of low rMSSD and HF in HRV. In constant with these findings, a previous study showed that LPA and mild-to-moderate OPA decreased the likelihood of a myocardial infarction (MI), while heavy OPA was not associated with a reduced risk [5]. We showed an increased risk of HRV from OPA, especially in aging workers. Whereas the previous study assessed OPA using subjective questionnaire, we used an objective measure of RHR. However, reports from the previous study that increasing OPA from mild to heavy elevates the risk for MI is in line with the findings from the current study (statistically significant in the model I adjusted for age, sex, and socio-economic status).

As rMSSD and HF are considered a reflection of vagal outflow [21, 24], high OPA might causes a repression of parasympathetic nervous system (PNS) activity. Decreased PNS activity has been explained by a gradual atrophy of the system due to cumulative fatigue [25], and it is widely established that HRV decreases with age [26]. Previous studies on the effects of exercise on HRV show differing results according to activity level. Moderate exercise increases HRV HF due to cardiovascular adaptation to aerobic training [27]. On the contrary, long term physical overtraining decreases rMSSD, LF, and HF [28]. In the current study, exercise has beneficial effects only in the younger group. Previous studies have also shown different beneficiary effects of exercise on HRV according to age, with younger individuals showing a greater increase of HRV after training [27]. Thus this study shows that high OPA has similar effects as over-exercising with the adverse effects of OPA in old age possibly explained by a gradual exhaustion of PNS activity and loss of cardiovascular adaptation.

In current study, social support and shift work were independent occupational risk factors on HRV. Social support can act as a buffer, improving adaptation to changing and stressful situations [29]. Conversely, altered circadian rhythm from night shift work can cause adverse changes in HRV [30, 31]. One of the most interesting finding from this study is that all older workers with high OPA exercise regularly. This may be explained in several ways, one of which may be the small number of participants over the age of 45. An alternative explanation is the healthy survival effect, in which older individuals who work in strenuous OPA conditions needs to build up physical fitness in order to survive in the workplace, while physically weaker workers would have left the high OPA work job. If the latter explanation is correct, measures should be taken to protect aging workers in physical and social contexts.

This study has several limitations. Causal relationships are difficult to determine from cross sectional study design. In addition, the number of study subjects included is too small to evaluate all of the original hypotheses, especially for the effects of high OPA in relation to low exercise in the older age group. Further, although we used objective marker to check OPA, we were not able to use objective tools to evaluate LTA, which could have resulted in a misclassification of LTA. Well-designed longitudinal studies with larger samples using objective measures of OPA and LTA are needed to examine the remaining questions. Although the techniques of HRV have been standardized, there are many problems to solve [21]. Five minute methods are strength to evaluate in a short time, while it is less accurate than 24 h method. Also there are intra-personal variations according to diurnal rhythm, breathing status, coffee, and smoking. Also there still have some grey area of normality and interpretation among time and frequency domains [21, 22]. Although we tried to reduce those problems including restriction of evaluation time and atmosphere, coffee and smoking, those limitation might influence results. Although we evaluated OPA with objective methods, we could not fully evaluate work characteristics among 5 occupational groups. Because detailed work characteristics might be different between groups, the objective OPA might have been misclassified in some degree.

Despite these limitations, this study revealed adverse effects of high OPA in older age workers. Prolonged working hour and excessive OPA are major occupational problems in Korea, with corresponding increases in the compensable CVD [32]. As adverse HRV is a preclinical biomarker for CVD including coronary heart disease, stroke, dysrhythmia, and sudden death [2, 21], the findings of this study potentially indicate that high OPA could cause CVD. This study showed adverse effects of high OPA on health by examining work environment using RHR and working hours to calculate OPA, as suggested by a prior investigation [20]. The results of this study suggest the need for reducing physical work load, or the number of working hours if work load can not be reduced, according to the work limit suggested in the previous study. These findings are especially important for workers over the age of 45. This study also indicates the need for further research into optimal OPA in workers.

## Conclusions

This study showed adverse effects of high OPA on health by examining work environment using RHR and working hours to calculate OPA. The results of this study suggest the need for reducing physical workload, or the number of working hours if workload cannot be reduced, according to the occupational exposure limit. These findings are especially important for workers over the age of 45. This study also indicates the need for further research into optimal OPA in workers.
